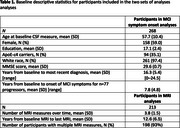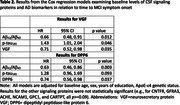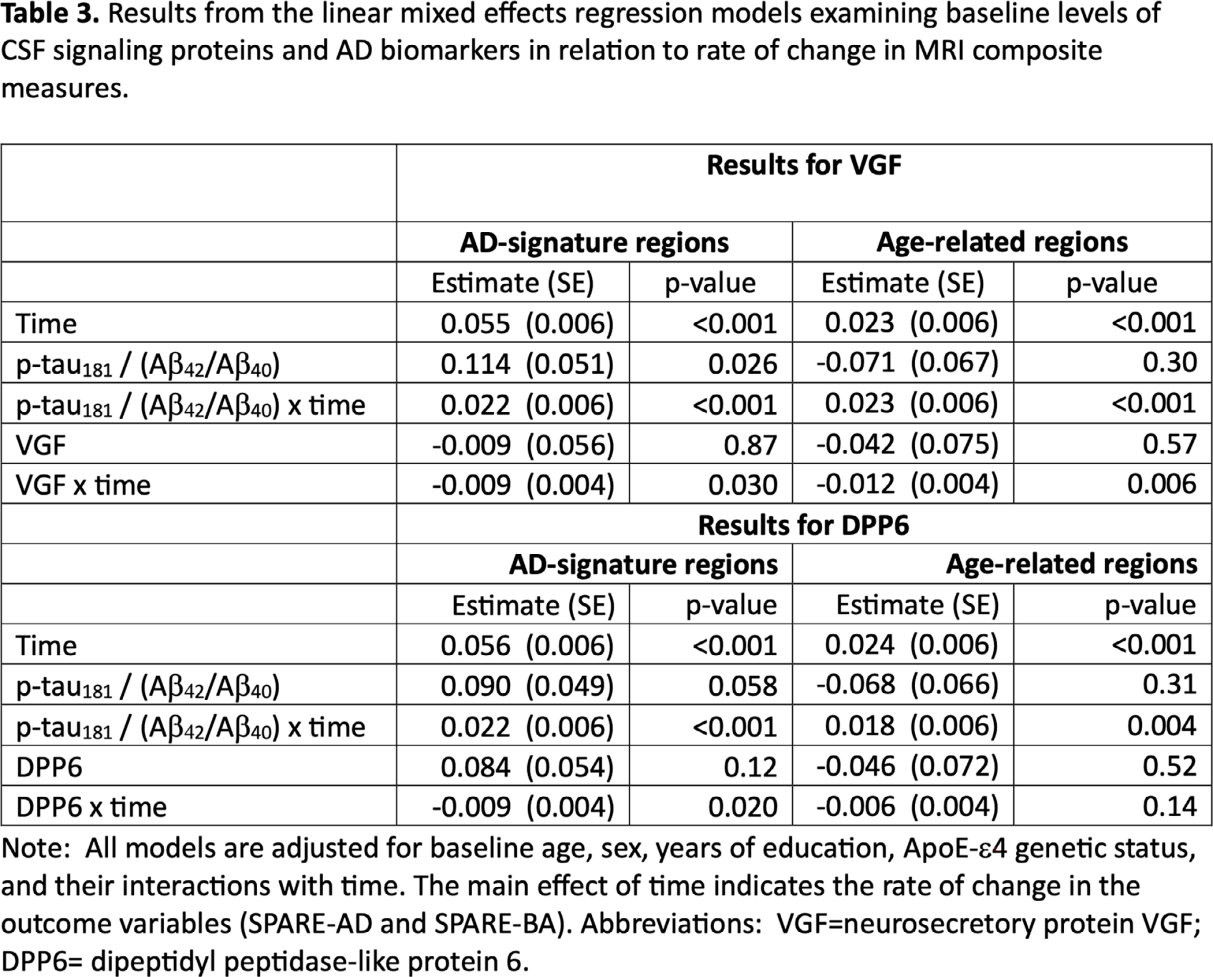# CSF synaptic proteins are associated with risk of MCI symptom onset and long‐term brain atrophy

**DOI:** 10.1002/alz70856_105326

**Published:** 2026-01-07

**Authors:** Anja Soldan, Chan‐Hyun Na, Yuxin Zhu, Corinne Pettigrew, Abhay Moghekar, Guray Erus, Christos Davatzikos, Marilyn S. S. Albert, Paul F Worley, the BIOCARD Study Team

**Affiliations:** ^1^ Department of Neurology, Johns Hopkins University School of Medicine, Baltimore, MD, USA; ^2^ Johns Hopkins University School of Medicine, Baltimore, MD, USA; ^3^ Johns Hopkins Bloomberg School of Public Health, Baltimore, MD, USA; ^4^ Centre for Biomedical Image Computing and Analytics, University of Pennsylvania, Philadelphia, PA, USA; ^5^ Perelman School of Medicine, University of Pennsylvania, Philadelphia, PA, USA

## Abstract

**Background:**

More abnormal cerebrospinal fluid (CSF) levels of amyloid and tau among cognitively unimpaired individuals are associated with higher risk of mild cognitive impairment (MCI) or dementia due to Alzheimer's disease (AD). However, the predictive accuracy of these markers alone is limited. This study examined whether novel synaptic markers and neural signaling proteins are associated with time to onset of MCI symptoms and with long‐term neurodegeneration, based on MRI, after accounting for traditional CSF AD biomarker levels.

**Method:**

CSF was collected from 268 cognitively unimpaired BIOCARD Study participants (mean baseline age = 57.7 years; mean follow‐up = 16.3 years; n = 77 progressed to MCI/dementia; see Table 1). Levels of eight peptides involved in neuronal signaling were measured, using quantitative parallel reaction monitoring mass spectrometry: CNTFR, GFRA3, VGF, ACHE, NCAM1, DPP6, GPC1, and CARTPT. Levels of Aβ_42_/Aβ_40_ and *p*‐tau_181_ were measured from the same CSF specimens using Lumipulse assays. Atrophy on volumetric MRI scans was quantified from 213 participants as two composites: (1) AD signature regions (SPARE‐AD) and (2) regions indexing brain aging (SPARE‐BA). All analyses covaried age, sex, years of education, and APOE‐e4 status.

**Results:**

In Cox regression models, after accounting for covariates and baseline AD biomarker levels, higher baseline levels of both neurosecretory protein VGF and dipeptidyl peptidase‐like protein 6 (DPP6) were associated with a lower risk of MCI symptom onset (both *p* <0.04, see Table 2. In mixed effect models, higher levels of VGF and DPP6 were also associated with less atrophy over time in AD‐signature regions (see Table 3), and higher VGF was associated with less age‐related atrophy. Interestingly, when AD biomarker levels were not accounted for, none of the signaling proteins were associated with MCI symptom onset or MRI atrophy.

**Conclusion:**

These findings suggest that among cognitively unimpaired individuals, higher levels of the synaptic proteins VGF and DPP6 may slow the onset of the symptomatic phase of AD as well as longitudinal atrophy in AD‐vulnerable regions, independently of AD pathology levels. These findings support the view that VGF and DPP6 may be resilience factors against neurodegeneration and cognitive decline and represent fruitful targets for novel therapeutics.